# Robotic Plaster Spraying: Crafting Surfaces with Adaptive Thin-Layer Printing

**DOI:** 10.1089/3dp.2020.0355

**Published:** 2022-06-09

**Authors:** Selen Ercan Jenny, Ena Lloret-Fritschi, David Jenny, Eliott Sounigo, Ping-Hsun Tsai, Fabio Gramazio, Matthias Kohler

**Affiliations:** Department of Architecture, Chair of Architecture and Digital Fabrication, ETH Zurich, Zurich, Switzerland.

**Keywords:** robotic plaster spraying, adaptive fabrication, thin-layer printing, visualization of material behavior, data-driven prediction models

## Abstract

Embedded in a long tradition of craftsmanship, inside or outside building surfaces, is often treated with plaster, which plays both functional and ornamental roles. Today, plasterwork is predominantly produced through rationalized, time-, and cost-efficient processes, used for standardized building elements. These processes have also gained interest in the construction robotics field, and while such approaches target the direct automation of standardized plasterwork, they estrange themselves from the inherent qualities of this malleable material that are well known from the past. This research investigates the design potentials of robotic plaster spraying, proposing an adaptive, thin-layer vertical printing method for plasterwork that aims to introduce a digital craft through additive manufacturing. The presented work is an explorative study of a digitally controlled process that can be applied to broaden the design possibilities for the surfaces of building structures. It involves the spraying of multiple thin layers of plaster onto a vertical surface to create volumetric formations or patterns, without the use of any formwork or support structures. This article describes the experimental setup and the initial results of the data collection method involving systematic studies with physical testing, allowing to develop means to predict and visualize the complex-to-simulate material behavior, which might eventually enable to design with the plasticity of this material in a digital design tool.


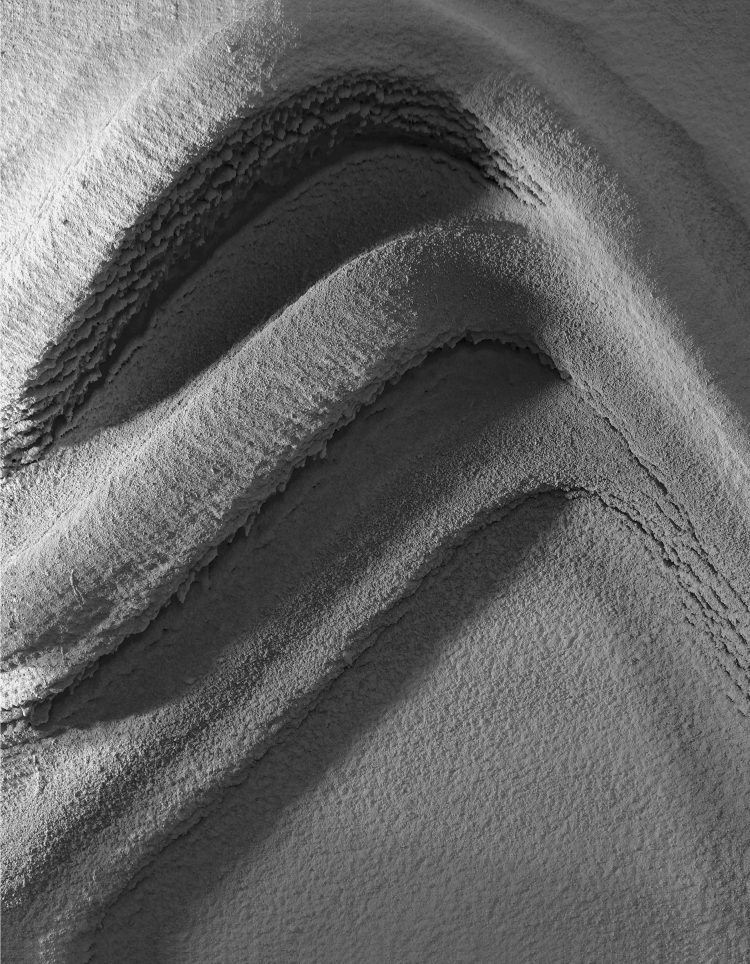


## Introduction

**FIG. 1.**
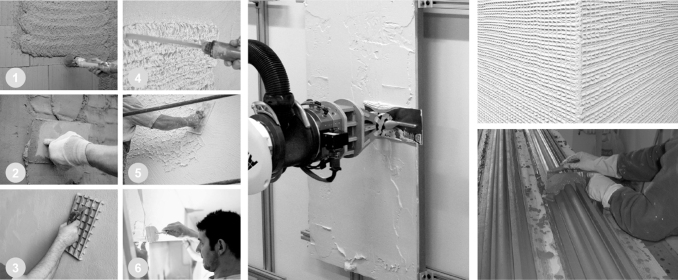
*Left*: Steps of a typical plastering process: (1), (2), (3) application of the base coat; (4), (5) application of the top coat; and (6) application of the smooth coat. *Middle*: Robotic arm with a smoothening trowel, imitating one step of the manual procedure, Bard *et al.*^2^ *Top right*: Textural detail from surface finishing, Spiro *et al.*^1^ *Bottom right*: Plasterer with a running mold, taken from https://www.plasterworkspecialist.com.

The inside or outside surfaces of building structures are often treated with materials such as cement, lime, or gypsum plaster that can have both functional and ornamental roles. In general, the functional role is to protect the building structure, or to improve the acoustic performance and thermal properties. The ornamental roles relate to the production of aesthetic qualities and variations to the surfaces of the built structure, although this is often neglected in current practice, as described in *Über Putz: Oberflächen entwickeln und realisieren* by Spiro *et al.*^1^ The application of plaster to interior walls and ceilings, as well as to façades, is a craft that requires specific tools, intuition, and a particular skill set. It is a challenging process that is carried out in several steps and in consecutive layers,* as shown in Figure 1 (left, images 1–6).

The challenges have also been addressed by the construction robotics field and led to the early attempts in the 1990s to replace the manual plastering process with automation approaches. These initial attempts, and similar approaches conducted since then, aim to apply plaster by using a multiple degrees of freedom (DoF) robotic arm, as shown in Figure 1 (middle). They seek to imitate the steps of a simplified plasterwork with the aim to introduce a time- and cost-efficient process for producing standardized, flat surfaces. In such a direct automation approach, the architectural potentials of exploring the three-dimensionality of plaster with a digitally controlled fabrication process are often neglected. However, using the DoF of an agile robotic arm for plasterwork could possibly address a digital crafting process that reinterprets the dexterity and versatility offered by the craftsmanship of the past (Fig. 1, right).

This research proposes a robotic plaster spraying process, referred to as “Robotic Plaster Spraying” (*RPS*), which addresses the challenge of sensing and control for adaptive spray-based printing, aiming to expand the design space of surfaces of building structures. In contrast to conventional manual plastering approaches, which involve the application of centimeter-thick layers of plaster that are then shaped with tools or formwork (Fig. 1, right), the proposed additive manufacturing method involves the application of multiple, millimeter-thin, and adapting layers of plaster, which is then repeated to build up volumetric formations or textural patterns. The goal is to explore the design space of the material's unique properties through an adaptive printing process, while maintaining a high degree of control, and to explore the versatility of plastering with an expanded design freedom.

### Relevant work

To facilitate an on-site *RPS* process, a mobile construction robot must be able to localize itself, both globally in reference to an absolute coordinate system and locally in reference to the existing building elements, that is, walls, columns, or ceiling, to allow the task to be executed.^3^ The problem of how to manage the flow of data between a plastering robot on-site and a building model, to enable adaptive, bespoke fabrication is an emerging topic.^4–6^ Early construction robotics research dating back to the 1990s, such as the interior finishing robot (spraying and tile setting) *TAMIR*^7^ or the autonomous plastering robot for walls and ceilings,^8^ demonstrated the feasibility of a time- and cost-efficient approach to the production of standardized surfaces. However, they lacked the technological means to develop an adaptive fabrication process to apply material informed plasterwork to building elements.

The construction robotics start-up OKIBO^†^ has recently demonstrated a mobile construction robot with integrated sensing capabilities that can be deployed for an adaptive, on-site wall plastering process. However, their approach, similar to the attempts in the 1990s, is focused on increasing productivity, imitating the steps of simplified plasterwork with the intention of introducing an efficient automated process for the production of standardized, flat surfaces. In other words, it does not fully exploit the architectural potentials of combining the three-dimensionality of plaster with an adaptive fabrication process.

**FIG. 2.**
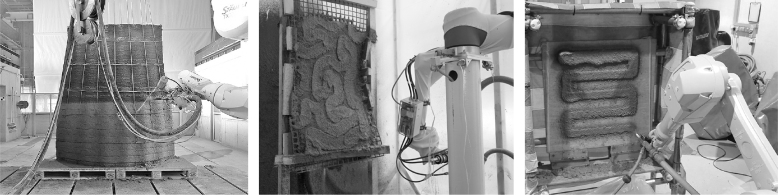
*Left*: Shotcrete 3D Printing, investigating freeform concrete elements with high surface qualities, Hack and Kloft.^9^ *Middle*: Robotic *AeroCrete,* a novel robotic spraying technology for slender, bespoke concrete elements, Taha *et al.*^10^ *Right*: S-3DCP research investigating the effect of process parameters on material distribution in a spray-based printing process, Lu *et al.*^11^

Nevertheless, there is an emerging field of research in which the focus is on adaptive, continuous fabrication processes that use malleable materials for the production of bespoke architectural elements, through spraying or printing. Some recent examples in this field are Shotcrete 3D Printing^9^ (Fig. 2, left) and *AeroCrete*^10^—a novel robotic spraying technology for the production of slender, bespoke concrete elements (Fig. 2, middle). Another example can be found in the S-3DCP research by NTU,^11^ which investigates the effect of process parameters on material distribution in a spray-based 3D concrete printing process (Fig. 2, right) for functional coatings in the form of overhanging applications on facades and ceiling decorations. It investigates the development of an analytical model to understand material behavior for guiding the selection of suitable parameters for the desired spray width and thickness through the quantitative deposition of the material on the target surface.

While the above projects explore material behavior and surface qualities to a certain extent, they lack the means to inform the design process, before fabrication. Even though there are different approaches to the simulation of cementitious materials in general, the majority of research does not focus on the most critical phase for digital fabrication: the transition from liquid to solid, which occurs in the order of multiple seconds to minutes, and provides substantial data for informing the design process on material behavior. Instead, most studies address the issue either from the point of view of cement hydration or strength development at the order of days.^12,13^ As such, there are currently no efficient means to design with the physical properties and the fabrication constraints of such complex, malleable material systems using digital tools. Most simulations rely on a numerical methodology, rather than a verification of models by comparing them with physical experiments.^14^ As such, neither the adaptive, continuous mobile fabrication systems, such as OKIBO, nor previous studies on simulation of complex materials put forward a method for the exploration of the design potentials of plaster or other malleable material systems targeting diverse surface qualities.

However, in recent years, research in digital fabrication has aimed to tackle the issue of the prohibitive complexity of simulation by developing data-driven design tools to explore the design potentials of specific processes with a relative precision in the prediction, driven by sensor systems that are becoming more precise and easily accessible.^15–18^ One current example in that regard is the research project Spatial Wire Cutting,^19^ where the complexity for the prediction of the fabrication process comes from the respective interaction of a loose and form-adaptive hot-wire adapting itself against the resistance of the processed material. In this research project, data from the fabrication parameters such as heat input, cutting speed, and forces are aligned with the resulting geometries. This technique allows controllable physical factors of the process to be correlated and inserted into the design generation, resulting in a data-driven design tool for cutting foam. A similar approach is used for Adaptive Robotic Carving,^20^ where sensors are utilized to record a person while carving, including all specific forces and movements required. Then, the collected data are used as input for a material and fabrication aware design process. Both projects do not target to fully understand material behavior at the granular level. Instead, they rely on a marginal understanding of material behavior in response to the needs of the fabrication system, without depending on a complete and detailed model description.

The research project presented in this article adopts the approach of recording fabrication parameters and material behavior in the process of making and converting those actions into a prediction tool that relies on linear and nonlinear regression models.

### Robotic plaster spraying

**FIG. 3.**
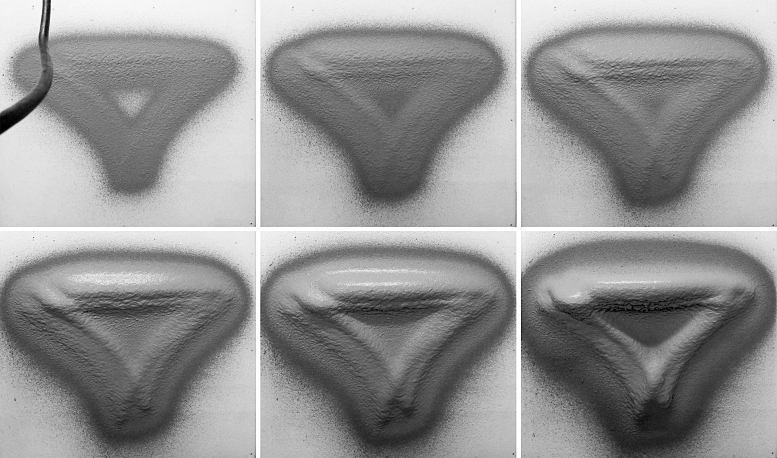
*RPS* process building up a volumetric formation, with adaptive thin-layer printing on a vertical surface. Result of 35 layers with spraying velocity varying between 0.025 and 0.2 m/s, and with a spraying distance of 500 mm. Total fabrication time: ∼2 h, with ∼75 kg of plaster. Waiting time between consecutive layers: ∼30 s. Final thickness on target surface (overhang): ∼18.5 cm. *RPS*, robotic plaster spraying.

*RP*S explores the material informed design process of bespoke surfaces, combining an off-the-shelf, fast setting cementitious plaster mix with an adaptive, continuous mobile fabrication process. In this process, a 6-DoF robotic arm is used to spray plaster onto a target surface (Fig. 3) for data collection through physical testing. Data collection involves scanning of the target surface to store information on the volumetric or textural formation in line with the fabrication parameters. As such, the goal is the delivery of an intuitive digital tool that can support the design process with this malleable material. The research addresses the challenge of a design and visualization method for plasterwork, and interlinks the adaptive *RPS* process to target geometries. Eventually, *RPS* aims to be intuitively used by designers and craftsmen, on and off site, for exploring bespoke building elements.

### Setup and scope of the study

**FIG. 4.**
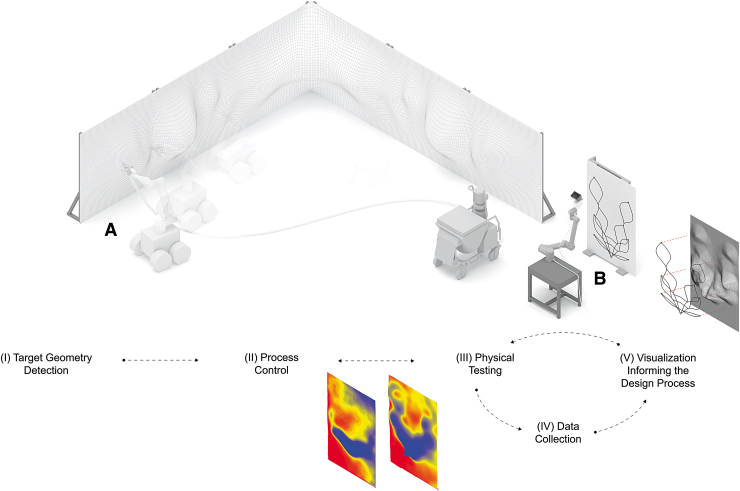
Diagram showing the components of the system, the overall setup, and the workflow: **(A)** Mobile testing setup. **(B)** Temporary stationary testing setup.

To validate the proposed method for *RPS*, five distinct components are being developed, as shown in Figure 4:

(I) Target geometry detection
(II) Process control
(III) Physical testing
(IV) Data collection
(V) Visualization informing the design process.

The first component addresses defining and interlinking the target geometries (i.e., the wall), on which *RPS* would be executed, with the mobile robot. A detailed description of the process is presented in Ercan Jenny *et al.*, “Online Synchronization of Building Model for Mobile Robotic On-Site Construction” (2020).^21^ The second component addresses process control through sensing and geometry acquisition for adapting the robot trajectories (spray paths) to maintain the selected values of the fabrication parameters. The trajectory is adapted after each spraying iteration, by projecting it onto the built state of the target surface for adjusting to the desired spraying distance, angle, and velocity, which is presented in Ercan Jenny *et al.*, “Crafting Plaster through Continuous On-Site Robotic Fabrication” (2020).^22^ In each iteration of the process, thin layers of plaster are sprayed, adapting to the material formation on the target surface.

This article describes the physical testing method, executed in a temporary stationary setup as a first step, that is being developed for data collection and for implementing a visualization tool informing the design process. These data are currently used to investigate the effect of different fabrication parameters, such as spraying velocity and distance (i.e., the effect on the thickness and the pattern of the plasterwork building up). The focus of this article, therefore, is components (III)–(V).

## Materials and Methods

**FIG. 5.**
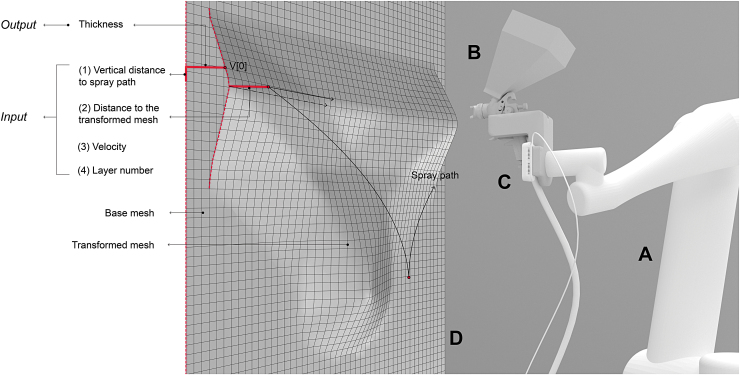
The overall (stationary) fabrication setup used in the tests. **(A)** 6-DoF robotic arm. **(B)** A robotically manipulated manual plastering spray gun. **(C)** An integrated *Intel RealSense Depth Camera D435i*. **(D)** A target spraying surface.

To reduce the complexity of the targeted on-site mobile *RPS* process, the investigation is divided into two different setups, as shown in Figure 4: (A) mobile and (B) stationary. The overall (stationary) fabrication setup used in the tests (shown in Fig. 5) comprises a (A) 6-DoF manipulator (collaborative robotic arm, *UR10*), (B) a robotically manipulated manual plastering spray gun, (C) an integrated *Intel RealSense Depth Camera D435i*, a *Hobart N50* mixer, and (D) a target spraying surface.

### Material system and spraying components

The tests presented in this article are carried out with the stationary setup (Fig. 4B). In this process, a base coat plaster, *Weber IP 18 Turbo,* is mixed using the *Hobart N50* mixer, and fed into the manual spray gun that is manipulated by the 6-DoF robotic arm. In the tests, the material flow (from the spray gun) is kept constant, while the velocity of the robotic arm, the spraying distance from the target surface, and the nozzle diameter of the plastering spray gun control the feed rate—thus determining the amount of plaster sprayed onto the target surface in each layer. Each spray path is iteratively repeated until an intended volume or pattern is achieved. To avoid sagging of the material, a waiting time (of ∼30 s) is introduced between the consecutive layers.

### Physical testing

Physical tests are carried out to gain empirical knowledge on the effect of the fabrication parameters on the material formation and they serve as the foundation for the data collection method. These tests are conducted in a systematic way, initially with simple spray paths (Table 1), with the intention of analyzing the material behavior in full-scale. Different values are chosen for the spraying velocity and distance, investigating the bounds of the parameters, maintaining the maximum single layer thickness of ∼5 mm, which ensures the material not to sag down from the target surface during buildup. Once the bounds are set, these values are explored within more complex designs (spray paths), hence revealing the design space of *RPS* and enriching the data collection, as presented in the Demonstrators section.

**Table 1.** Initial “Matrix” of Tests on Spraying Distance and Velocity, with Simple Curves, Representing Spray Paths

Spray path	Distance of spraying, mm	Velocity of spraying, m/s
*_______*	300	Constant velocity (0.75)
*_______*	400	Linear acceleration (0.3–1)
*_______*	500	Sinusoidal acceleration (0.3–1)

### Data collection

**FIG. 6.**
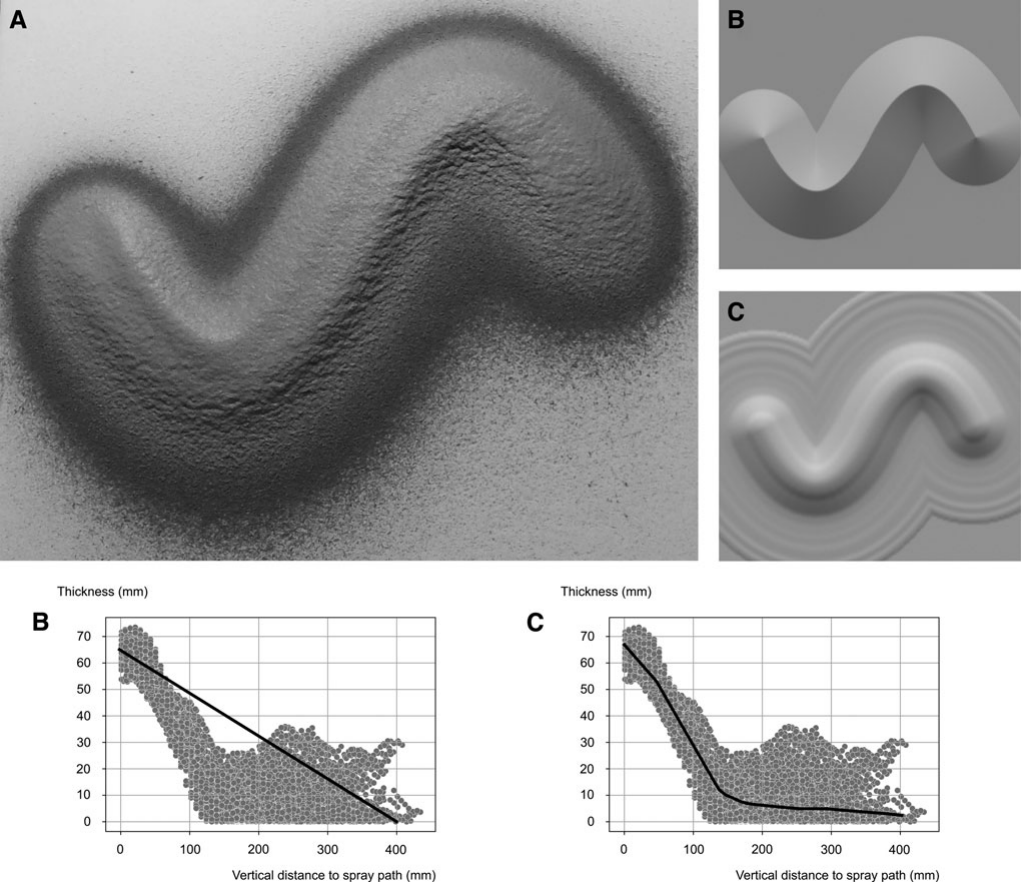
**(A)** Result of 15 layers, with a spraying velocity of 0.75 m/s and a spraying distance of 400 mm. **(B)** Visualization with the linear model. **(C)** Visualization with the nonlinear model.

The collected data permit the development of a suitable approach for storing the sensed surface geometry—the physical result—in an extended mesh data structure. The data, consisting of the mesh vertices and the fabrication parameters, are stored after each spraying iteration.^‡^ The goal is to use these data for visualizing the effect of the fabrication parameters in line with the material. The proposed method computes the transformation of the vertices of the target surface in two consecutive states (base mesh and transformed mesh, Fig. 5). The actual state of the target surface is recorded as a high-resolution quasiregular trimesh by the depth camera mounted on the spray gun (Fig. 5C). The base mesh (a lower resolution regular quad mesh) is then projected onto this state and transformed (shown as a transformed mesh in Fig. 5). By computing this transformation after each spraying iteration (layer), the volumetric formation is tracked vertex-by-vertex.^§^ These recorded data are the base for establishing a digital (visualization) tool (Fig. 6) that supports the design process before fabrication. In this tool, both linear and nonlinear functional relationships are used between the parameters and the material formation, as explained in the Linear model and Nonlinear model sections.

### Visualization informing the design process – prediction models

The visualization tool is one of the key components for the research on *RPS*. The goal is to inform the designer on the combined effect of the fabrication parameters, such as spraying velocity and distance. Accordingly, it aims to enable the design of bespoke surfaces, while also providing fabrication data, such as the number of layers to be sprayed to achieve a specific geometry. For this, currently two different approaches are being investigated—a linear and a nonlinear model. In both models, for a given input—(1) the vertical distance to the spray path, (2) the end-effector distance to the transformed mesh, (3) the velocity of the trajectory, and (4) the layer number, representing each spray path—the output is computed and visualized as the sprayed plaster thickness (Fig. 5). Eventually, the goal is to provide an intuitive design tool that can predict and visualize the resulting surfaces from a particular spray path.

#### Linear model

It implements a linear function that establishes the relationship between the input and the output with a set of empirical data (that is recorded and measured during the initial tests, see the Effect of the fabrication parameters section). To this end, the derived minimum and the maximum values are mapped to the output (sprayed thickness), giving an approximate representation of the possible outcome and an empirical understanding of the parameters and their effects. It is calculated as follows:


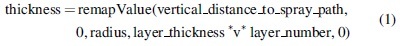


#### Nonlinear model

It uses a nonlinear regression solver, through Sequential Minimal Optimization, to predict the physical results of the spraying process. Each input/output (data) set is used for training, through LunchBox,** which allows for the exploration of nonlinear regression algorithms to compute the prediction, trained with the data recorded in the meshes (that are serialized to JSON). Through this model, untested conditions, such as multiple overlapping spray paths, are visualized, which are more complex to model using the linear function described above.

## Results and Discussion

The results are presented in two main sections. The first Effect of the fabrication parameters section describes the results of the physical tests using simple spray paths of the initial testing “matrix,” as shown in Table 1. The second Demonstrators section presents the preliminary usage of the visualization tool for the design of prototypes with more complex spray paths, and at a larger scale.

### Effect of the fabrication parameters

To investigate the effect of the fabrication parameters, the first “matrix” of tests involves a series of simple spray paths, with different values chosen for spraying distance and velocity (Table 1). These initial systematic tests are conducted to record, measure, and analyze the effect of the fabrication parameters on the physical results (material formation) for a fixed number of 15 consecutive layers of sprayed plaster. The results of this first series of tests form the basis of the data used in the visualization models, establishing the relationship between the fabrication parameters and the physical results. To reduce the complexity of the investigation, the spraying angle is fixed at a value that keeps the spraying tool orthogonal to the target surface.

#### Distance of spraying

The distance of spraying is tested in a range of 300–500 mm (Table 1). To maintain consistency, for all the tests involving distance, the velocity of spraying is kept at a constant value of 0.75 m/s. The results show that a spraying distance of 500 mm produces a spray radius of ∼250 mm with an ∼2 mm layer thickness, and a distance of 300 mm produces a radius of ∼150 mm with an ∼4 mm layer thickness on the target surface. These recorded data serve as the starting point for the function implemented for building the linear model (see the Linear model section) and for training the nonlinear function.

#### Velocity of spraying

The velocity of spraying is tested in a range of 0.3–1 m/s, with the three variations being (I) constant velocity, (II) linear acceleration, and (III) sinusoidal acceleration (Table 1). The goal is to explore the relationship between the volume of the plaster buildup on the target surface and the velocity of spraying. To ensure consistency, for all tests on velocity, the distance of spraying is kept at a constant value of 400 mm. Based on the recorded results, velocity is modeled with an inverse relationship to the layer thickness, and is implemented accordingly in the linear model (see the Linear model section). The recorded results are also included in the nonlinear model by training the function with the collected data.

### Demonstrators

The first demonstrator “Blending paths” aims at enhancing the effect of changing the velocity of spraying, giving the designer the choice of having the material built up in an amplified amount on selected (blended) portions of the spray paths. The second demonstrator, “The Wall,” aims at exploring surface qualities with varying textural patterns and volumetric formations, hinting at the extended design space of *RPS*.

#### Blending paths

**FIG. 7.**
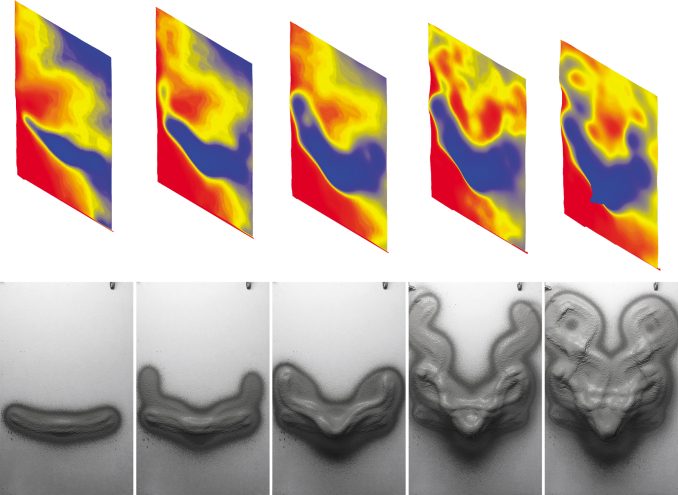
Fabrication steps (consecutive layers) of the “Blending paths” demonstrator, with the scanned state of the respective layers (*above*). Result of 72 layers with spraying velocity varying between 0.1 and 1 m/s, and with a spraying distance of 400 mm. Total fabrication time: ∼4 h, with ∼120 kg of plaster. Waiting time between consecutive layers: ∼30 s. Final thickness on target surface (overhang): ∼20 cm.

The design method implemented for “Blending paths” measures the distance between the input spray paths (curves) and uses these distances to determine the spraying velocity within a range of 0.1–1 m/s, while the spraying distance is kept at a constant value of 400 mm. The distance between paths (represented with curves) is measured at the points at which they are divided into equal length segments. The algorithm derives a lower velocity where a smaller distance between these length segments is measured, resulting in slower movement of the robotic arm where the curves are closer to each other. In this way, an accumulation of material buildup is achieved at locations where the curves blend in (Fig. 7). This algorithm is an early step in the creation of the user-designed “effects,” supported by the visualization tool that informs the design process, and enables an intuitive exploration of the design space of *RPS* (see the Visualization informing the design process – prediction models section).

#### The wall

**FIG. 8.**
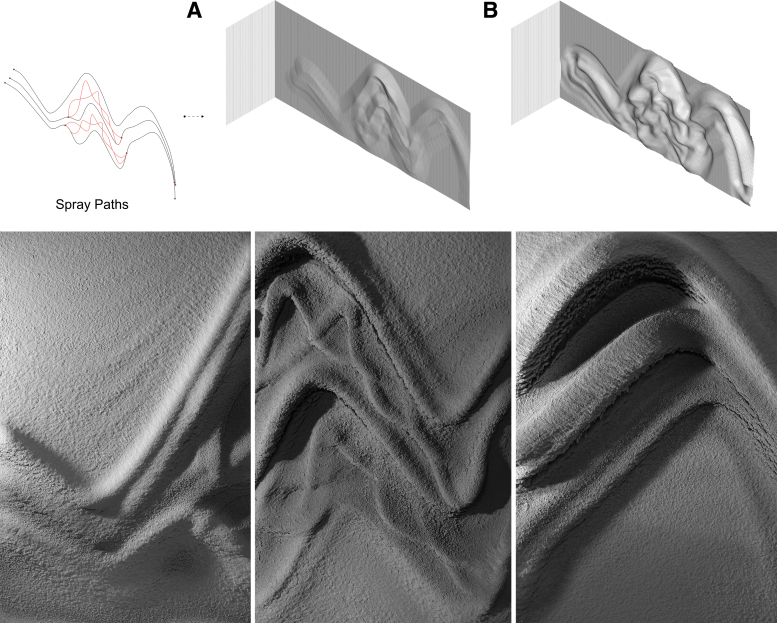
Design and fabrication of “The wall.” *Above*: Visualization of spray paths before fabrication with **(A)** linear model and **(B)** nonlinear model. Design consists of three main curves (spray paths), shown in *black*, and a second set of curves, shown in *red*. Result of ∼275 layers with spraying velocity varying between 0.1 and 1 m/s, and with spraying distance varying between 300 and 500 mm. Total fabrication time: ∼24 h, with ∼900 kg of plaster. Waiting time between consecutive layers: ∼30 s. Final thickness on target surface (overhang): ∼16.5 cm.

**FIG. 9.**
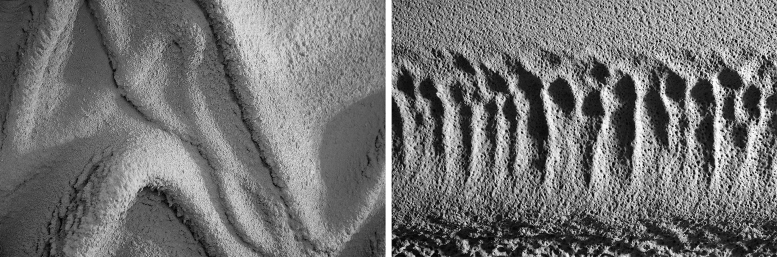
Detail views from the selected prototypes within the catalog of different surface geometries and textures produced with *RPS.*

This *demonstrator* aims at achieving the design effect of “Blending paths” on five panels measuring 1.75 m in height and 1 m in width, resulting in a wall with an overall length of 5 m. One segment of the panels is built as a corner to showcase the possibility of applying such a continuous fabrication process onto a building detail, where multiple elements come together. The spraying velocity ranges from 0.1 to 1 m/s, and the spraying distance ranges from 300 to 500 mm. The design consists of three main curves (spray paths, shown in black in Fig. 8), which expand along all the four panels with a volumetric formation that ends as a flat surface. Along these three main curves, a second set of curves is generated (shown in red in Fig. 8) to express various plastic qualities, hinting at the extended design space of plasterwork with *RPS* (Fig. 9). All spray paths are visualized using the tool described in the Visualization informing the design process – prediction models section, allowing the design to be refined before fabrication. The spraying time of this prototype is ∼24 h, and involves ∼900 kg of cementitious plaster.

## Conclusions and Next Steps

This article presents the first results of a data collection method that promises a novel visualization and design tool for *RPS*, which supports the understanding of complex material behavior of an off-the-shelf cementitious plaster mix in a digitally controlled fabrication process. The two models (linear and nonlinear) used for prediction show different potentials. The linear model is a simplistic interpretation of the fabrication parameters giving an approximate representation of the textural patterns or volumetric formations. As such, it allows for a quick estimation of results and can easily be adapted to changes in the fabrication parameters. The nonlinear model has the capability of representing a high level of detail, even with the limited amount of data provided at the current state of the research (Fig. 6). While the limited data collected with the current sensing system, especially regarding the details of the surface textures, the results promise the potential of the nonlinear model for visualizing untested conditions and complex designs with numerous overlapping spray paths.

Overall, the goal is to revisit the commonly known roles of plaster providing durability, insulation, and weather protection to the building structure while seeking the correlation to additional qualities of the material that provide visual, acoustic, or light diffusing effects through geometric complexity. For this purpose, a catalog of different surface geometries and textures is being developed (Fig. 9). However, the current sensing method is limited to retrieving the surface geometry to collect data to visualize the effect of the process parameters. As a next step, the sensing system will be extended to, a high-accuracy time-of-flight camera*,* to facilitate data collection on the quality of the target surface in a higher resolution. This will allow retrieving the details of the surface textures based on, reflectance to assess the surface smoothness and roughness. Further studies will explore the possibility of using denser meshes in data collection addressing the material roughness and surface texture in addition to the overall volumetric geometry.

**FIG. 10.**
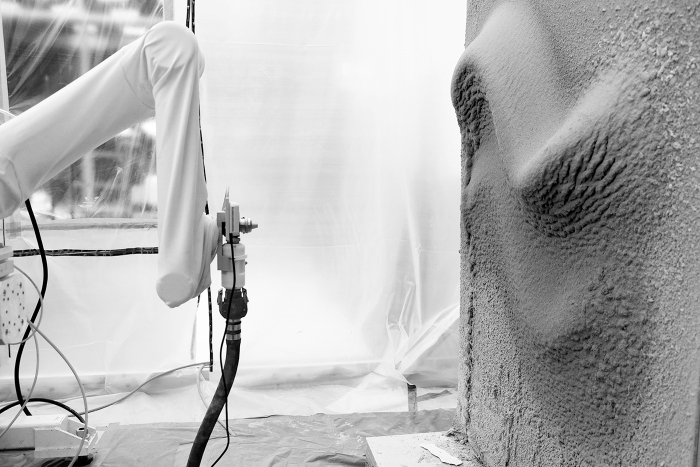
Preview from a further iteration of the fabrication setup, with an automated plaster spraying tool integrated.

Furthermore, material processing will be developed into a fully automated spraying and pumping system (Fig. 10), improving the robustness of the system, and to ensure the consistency of the collected data. In such a setup, further studies will be conducted using both the linear and the nonlinear models. The studies will include data collection for the quantitative analysis of surface qualities in-line with the fabrication parameters such as angle of spraying, in addition to velocity and distance. In addition, the influence of physical parameters such as gravitational force, as well as the properties of the material mix, will be empirically analyzed and included in the models. As such, the design process will be informed on the combined effect of all the parameters, for fully exploring the design space of the proposed thin-layer vertical printing process.

The overarching goal of *RPS* is eventually to develop a digital design and fabrication system for robotic spraying that is intuitive to use for designers and craftsmen by predicting the physical outcome, for which the presented preliminary work has been key subject to this article. For this research to have a meaningful impact, it is important to back-integrate production constraints and possibilities into a design and visualization tool, which poses a scientific challenge. However, such a tool can open up a design space of complex-to-simulate material processes and eventually can be applied to other materials and processes that go beyond plaster and save materials, making bespoke structure and surfaces more cost efficient to produce. In terms of on-site, adaptive execution of the continuous *RPS* of building elements, further tests will be conducted using the mobile setup. In this way, crucial details, such as corners where multiple building elements come together, will be included in the investigation, and the full potential of *RPS* will be showcased within an extended workspace that is not limited to the footprint of the robot base. Finally, the research promises transferring of the developed methods to other materials used for plasterwork such as clay, gypsum or lime. Like this, *RPS* aims to make a novel contribution to the field of additive manufacturing and to permit a deeper exploration of the surfaces of architectural spaces, enhancing the bespoke design potential of plaster with a new digital craft.

## Notes

^*^
A typical plastering process consists of (1) spraying of the base coat, that can be a lime and cement mix, (2) smoothing and leveling of the base coat, (3) scratching or scraping of the base coat, (4) spraying of the top coat, that can be a lime and white cement mix, (5) troweling of the top coat, and finally (6) application of the smooth coat, that can be a gypsum and lime hydrate mix.

^†^

https://okibo.com/

^‡^
The data are stored by serializing the mesh to JSON.

^§^
A lower resolution regular quad mesh is used as the base providing a grid to compute the transformation of each vertex, thus predicting the change in thickness. This allows to retain a clean mesh structure with persistent topology for the prediction model, and a fast computation time. The resolution of the mesh is defined by the number of vertices and is currently adapted globally. For further iterations of the workflow, we plan to implement a selective mesh subdivision approach adapting the mesh resolution locally to specific features of the overhanging or underhanging surfaces (such as high curvature, dense texture).

^**^
LunchBox is a plug-in for Rhino Grasshopper that allows for exploring general machine learning implementations such as regression analysis in addition to other functionalities.
